# Endothelial mechanisms for inactivation of inflammation-induced hyperpermeability

**DOI:** 10.1152/ajpheart.00543.2022

**Published:** 2023-03-03

**Authors:** Prerna R. Nepali, Pía C. Burboa, Mauricio A. Lillo, Patricio E. Mujica, Toru Iwahashi, Jihang Zhang, Ricardo G. Durán, Mauricio Boric, Nikola Golenhofen, David D. Kim, Natascha G. Alves, Andrew P. Thomas, Jerome W. Breslin, Fabiola A. Sánchez, Walter N. Durán

**Affiliations:** ^1^Department of Pharmacology, Physiology and Neuroscience, Rutgers New Jersey Medical School, Newark, New Jersey, United States; ^2^Department of Pharmacology, Physiology and Neuroscience, School of Graduate Studies, Rutgers, The State University of New Jersey, Newark, New Jersey, United States; ^3^Department of Natural Sciences, School of Health and Natural Sciences, Mercy College, Dobbs Ferry, New York, United States; ^4^Institute for Anatomy and Cell Biology, Ulm University, Ulm, Germany; ^5^Department of Molecular Pharmacology and Physiology, Morsani College of Medicine, University of South Florida, Tampa, Florida, United States; ^6^Facultad de Medicina, Instituto de Inmunología, Universidad Austral de Chile, Valdivia, Chile

**Keywords:** endothelium, hyperpermeability, inflammation, vascular biology

## Abstract

Microvascular hyperpermeability is a hallmark of inflammation. Many negative effects of hyperpermeability are due to its persistence beyond what is required for preserving organ function. Therefore, we propose that targeted therapeutic approaches focusing on mechanisms that terminate hyperpermeability would avoid the negative effects of prolonged hyperpermeability while retaining its short-term beneficial effects. We tested the hypothesis that inflammatory agonist signaling leads to hyperpermeability and initiates a delayed cascade of cAMP-dependent pathways that causes inactivation of hyperpermeability. We applied platelet-activating factor (PAF) and vascular endothelial growth factor (VEGF) to induce hyperpermeability. We used an Epac1 agonist to selectively stimulate exchange protein activated by cAMP (Epac1) and promote inactivation of hyperpermeability. Stimulation of Epac1 inactivated agonist-induced hyperpermeability in the mouse cremaster muscle and in human microvascular endothelial cells (HMVECs). PAF induced nitric oxide (NO) production and hyperpermeability within 1 min and NO-dependent increased cAMP concentration in about 15–20 min in HMVECs. PAF triggered phosphorylation of vasodilator-stimulated phosphoprotein (VASP) in a NO-dependent manner. Epac1 stimulation promoted cytosol-to-membrane eNOS translocation in HMVECs and in myocardial microvascular endothelial (MyEnd) cells from wild-type mice, but not in MyEnd cells from VASP knockout mice. We demonstrate that PAF and VEGF cause hyperpermeability and stimulate the cAMP/Epac1 pathway to inactivate agonist-induced endothelial/microvascular hyperpermeability. Inactivation involves VASP-assisted translocation of eNOS from the cytosol to the endothelial cell membrane. We demonstrate that hyperpermeability is a self-limiting process, whose timed inactivation is an intrinsic property of the microvascular endothelium that maintains vascular homeostasis in response to inflammatory conditions.

**NEW & NOTEWORTHY** Termination of microvascular hyperpermeability has been so far accepted to be a passive result of the removal of the applied proinflammatory agonists. We provide in vivo and in vitro evidence that *1*) inactivation of hyperpermeability is an actively regulated process, *2*) proinflammatory agonists (PAF and VEGF) stimulate microvascular hyperpermeability and initiate endothelial mechanisms that terminate hyperpermeability, and *3*) eNOS location-translocation is critical in the activation-inactivation cascade of endothelial hyperpermeability.

## Graphical abstract

**Figure:**
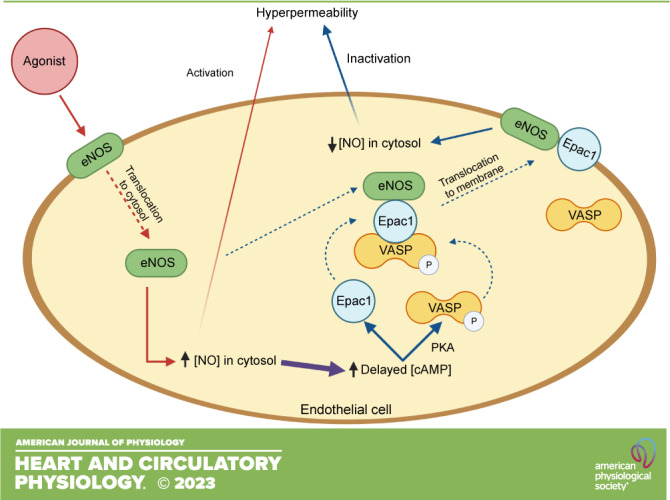

## INTRODUCTION

Microvascular hyperpermeability is a hallmark of the acute inflammatory response to tissue injury. The microvascular endothelium is the main regulator of this process. We and others have identified key regulatory mechanisms of the onset of microvascular permeability. Proinflammatory mediators trigger the beginning of hyperpermeability by activating signaling pathways that converge on endothelial nitric oxide (NO) synthase (eNOS) internalization to cytosol and cytosolic NO production (1–11). The restoration of endothelial barrier function following acute hyperpermeability is essential for vascular and tissue homeostasis since prolonged microvascular hyperpermeability may lead to edema, compartment syndrome, and organ failure. We propose that this restoration is an active process and define it as inactivation of hyperpermeability.

Classically, restoration of endothelial barrier function has been understood as a passive consequence of removing the proinflammatory agonist. However, there is evidence that agonist-induced hyperpermeability declines to normal baseline in vivo even in the continuous presence of the test agonists (1). Similarly, we reported that Iloprost (analog of prostacyclin, i.e., a vasodilator), administered at the time of reperfusion, inhibits the inflammation-associated increase in permeability caused by ischemia-reperfusion in striated muscle (12). This apparent departure from the classically accepted observation that vasodilators contribute to increases in microvascular permeability was explained subsequently in molecular terms by the report that Iloprost stimulates Rap1 (13). The distinct signaling pathway involving endothelial cyclic AMP (cAMP), its effector exchange protein activated by cAMP (Epac1) and Rap1 has been reported as an important mediator of endothelial barrier integrity (14–16). Moreover, stimulation of the endothelial cAMP-Epac1-Rap1 axis inactivates ischemia-reperfusion-induced hyperpermeability (14), highlighting its therapeutic potential. Therefore, we anticipate that cAMP is an important player in inactivation of hyperpermeability. Because eNOS location is fundamental for the onset of hyperpermeability (4–8), we also anticipate that eNOS location and NO production would be essential factors in inactivation of hyperpermeability. However, whether the pathways that regulate the onset (eNOS/NO) and the inactivation (cAMP/Epac1/Rap1) of hyperpermeability are functionally interacting mechanisms is unknown.

Based on the above-mentioned observations, we propose the novel hypothesis that inactivation of microvascular hyperpermeability is an active signaling process intrinsic to endothelial cells (EC) and is triggered by the agonist(s) causing hyperpermeability. Our hypothesis indicates that the eNOS/NO pathway and the cAMP/Epac1/Rap1 pathway are interacting mechanisms in the inactivation of agonist-induced microvascular hyperpermeability. We tested our hypothesis using platelet-activating factor (PAF) as the main proinflammatory agent, and either vascular endothelial growth factor (VEGF) or thrombin as secondary agonists. Our hypothesis states that the agonist triggers a delayed signaling cascade leading to inactivation of hyperpermeability. We examined this statement by measuring baseline and stimulated endothelial permeability as an end point before and after selective activation of Epac1 and/or agonist stimulation of cAMP production. The hypothesis was tested in vivo in the mouse cremaster muscle as well as in the hamster cheek pouch, and in vitro in human microvascular endothelial cells as well as in mouse microvascular endothelial cell lines from myocardium (MyEnd). We report evidence in support of our novel hypothesis.

## METHODS

### Animals

We used male and female wild-type mice (C57BL/6J, 30–40 g, Jackson Laboratories, Bar Harbor, MA) for the assessment of permeability in the cremaster muscle and male/female golden Syrian hamsters (100–150 g) for the measurement of microvascular permeability in the cheek pouch. The Rutgers New Jersey Medical School Institutional Animal Care and Use Committee approved the experimental procedures involving the use of animals. Experiments were carried out following the NIH’s Guide for the Care and Use of Laboratory Animals.

#### Cremaster muscle.

Mice weighing 30–35 g were anesthetized with sodium pentobarbital (50 mg/kg ip). A tracheostomy was performed to facilitate spontaneous respiration. A catheter was introduced into the left external carotid artery, and arterial pressure was continuously recorded. Pressure measurements were interrupted at 30-min intervals for the collection of arterial blood samples for subsequent fluorometric analysis. The left external jugular vein was cannulated for the administration of fluorochrome and supplementary doses of anesthesia. The cremaster muscle was prepared for fluorescent intravital microscopy. The muscle was exposed through a scrotal incision, separated from the skin, and cleared of connective tissue. The cremaster was incised longitudinally. Vessels connecting the cremaster and epididymis were divided with thermocautery. The testis was placed in the abdominal cavity. The cremaster was splayed in a chamber that allowed superfusion of both the upper and lower surface of the cremaster muscle. After the surgical preparation, the cremaster was superfused with a bicarbonate buffer solution adjusted to pH 7.35, equilibrated with 95% N_2_-5% CO_2_, and maintained at 36°C. The composition of the buffer solution was (in mM) 131.9 NaCl, 4.7 KCl, 2.0 CaCl_2_, 1.2 MgSO_4_, and 18.0 NaHCO_3_.

#### Hamster cheek pouch.

Male Syrian hamsters *(Mesocricetus auratus)* weighing 60–110 g were anesthetized with pentobarbital sodium (65 mg/kg ip) and positioned on a heating mat regulated at 37°C. The right cheek pouch was prepared for fluorescent intravital microscopy and suffusate sampling. The hamster was placed on a Lucite board and mounted on a microscope. The pouch was suffused with bicarbonate buffer at a constant flow rate of 1 mL/min. The composition of the buffer was the same as used for the cremaster muscle. Suffusion was interrupted only for topical application of vasoactive agents.

#### Mouse mesentery.

Male and female mice (30–40 g) were anesthetized with pentobarbital sodium (50 mg/kg ip), and fluorochrome and supplementary doses of anesthesia were administered via the jugular vein. Midline abdominal incisions were made, and mice were placed in an intestinal bath in the prone position to position the terminal ileum in the chamber.

Observations were made with a Nikon Optiphot microscope equipped with an adjustable stage. An episcopic fluorescence Ploem attachment was employed for fluorescent microscopy. Epi-illumination was provided by a 50-W mercury lamp source in conjunction with the appropriate filters for fluorescein isothiocyanate (FITC).

### Assessment of Microvascular Permeability by Intravital Microscopy

#### Hamster cheek pouch preparation.

The microvascular response to PAF was studied by intravital microscopy in the cheek pouch of 10 golden Syrian hamster, as described previously (17, 18). Baseline and agonist-stimulated permeability were measured by integrated optical intensity (IOI) (19, 20).

#### Mouse cremaster preparation.

We prepared the mouse cremaster for intravital microscopy, as described previously (1). We used 53 mice, including experiments shown in supplemental materials. We measured interstitial concentration of intravenously infused FITC-Dx70 before and after topical application of the agonist.

### Mouse Mesentery Preparation

We prepared the mouse mesentery, as previously described (1). Microvascular permeability under baseline and agonist-stimulated conditions in male and female mice was measured by IOI.

### Endothelial Monolayer Permeability Assay

In vitro endothelial permeability assays were carried out, as described previously (2–5, 21).

### Endothelial Resistance Assay

Transendothelial electrical resistance (TER) was determined using the electrical cell-substrate impedance sensing (ECIS) technique (22), using flow arrays and a pump system that allows washin and washout of media (23).

### Reagents

Platelet-activating factor-16 (1-*O*-palmityl-2-acetyl-*sn*-glycero-3-phosphocholine, PAF), thrombin, forskolin, fluorescein isothiocyanate (FITC)-labeled dextran-molecular weight 70,000 Da (FITC-Dx70), *N*^G^-monomethyl-l-arginine (l-NMMA), (E)-2-(5-(tert-butyridinezol-3-*yl*)-*N*′-(3-chlorophenyl)-2-oxo-aceto-hydrazonoyl cyanide (ESI-09) and 1-(*N,N*-dimethylcarbamoyl)-4-ethynyl-3-{3-fluoro-4-[(1H-2-methylimidazo[4,5yridinedin-1-*yl*)methyl]benzoyl}-indole, HCl (ABT-491) were obtained from Millipore Sigma (St. Louis, MO). 8-(4-Chlorophenylthio)-2′-*O*-methyladenosine-3′-5′-cyclic monophosphate sodium salt (8cPT-cAMP) was purchased from Tocris (Minneapolis, MN). Recombinant human vascular endothelial growth factor-165 (VEGF) was obtained from R&D Systems (Minneapolis, MN).

### Antibodies

Mouse anti-eNOS was from BD Transduction Laboratories (Franklin Lakes, NJ). Rabbit anti-VASP, anti-phospho-VASP (ser157), and anti-phospho-VASP (ser239) were from Cell Signaling Technologies (Danvers, MA). Goat anti-VE-cadherin was from Santa Cruz Biotechnology (Santa Cruz, CA). Mouse anti-β-actin was from Sigma-Aldrich (St. Louis, MO).

### cAMP Measurements

Intracellular cAMP was measured in confluent HMVEC monolayers using the direct cyclic AMP enzyme-linked immunosorbent assay (ELISA) kit (Enzo Life Sciences, Farmingdale, NY).

### Cell Culture, Transfection, and siRNA-Mediated Protein Depletion

Human dermal microvascular endothelial cells (HMVECs) and human umbilical vein endothelial cells (HUVECs) were from Lonza (Walkersville, MD). Cells were cultured in endothelial growth medium supplemented according to the manufacturer’s instructions. MyEnd cells (15) were cultured, as previously described (6). HMVECs were depleted of Epac1 or Mena/VASP with SMARTpool siRNA oligonucleotides from Dharmacon (Lafayette, CO). Briefly, 100 nmol/L siRNA mix were complexed with OligofectAMINE reagent (Thermo Fisher Scientific, Parsippany, NJ) and cells were exposed to the lipid-oligonucleotide mixture for 4–6 h. Transfection efficiency was evaluated by Western blotting.

### Immunocytochemistry and Imaging

Cells were grown on gelatin-coated glass coverslips and fixed for 30 min with 4% paraformaldehyde at 4°C immediately after treatment, and permeabilized with 0.1% Triton X-100 for 5 min. After blocking, primary antibodies were applied for 1–2 h at room temperature. Alexa Fluor-conjugated secondary antibodies were applied for 1 h at room temperature, and coverslips were mounted with ProLong Gold with DAPI mounting medium (Life Technologies). Samples were imaged using a Carl Zeiss AxioVert 200 M inverted epifluorescence microscope with an oil immersion 63 × 1.45 NA Apochromat objective. Micrographs were processed and linearly adjusted for contrast using AxioVision rel.4.8 software (Carl Zeiss) and composed using CorelDRAW Graphics Suite.

### Proximity Ligation Assay

The DuoLink In Situ PLA kit (Olink Biosciences, Uppsala, Sweden) was used to measure the association between Epac1 and eNOS. Proximity ligation assay (PLA) probes and secondary antibodies conjugated to antiparallel double-stranded DNA template oligonucleotides were used for visualization of Epac1 and eNOS proximity after primary antibody incubation.

### Statistical Analysis

Data are shown as means ± SE. Statistical analysis was performed using GraphPad Prism 6 software. Comparisons of single treatments were done using Student’s *t* test and one-way ANOVA followed by Tukey’s or Newman–Keuls post hoc test to determine the significance between groups. Multiple treatments within groups were assessed with repeated-measures ANOVA followed by Newman-Keuls or Tukey’s post hoc test. Significance was accepted at *P* < 0.05.

## RESULTS

### Endothelial Barrier’s Restoration Is Self-Limiting

Previous results have shown that proinflammatory agonists (histamine, bradykinin, PAF, VEGF, thrombin) promote an increase in endothelial barrier permeability (3, 11, 18, 21, 24–26). This phenomenon is transient in healthy conditions, which suggests that there is a mechanism associated with hyperpermeability inactivation. Therefore, we postulated the conceptually novel hypothesis that barrier restoration is a self-limiting active process that reflects endothelial mechanisms for inactivation of stimulated hyperpermeability.

**Figure 1.**
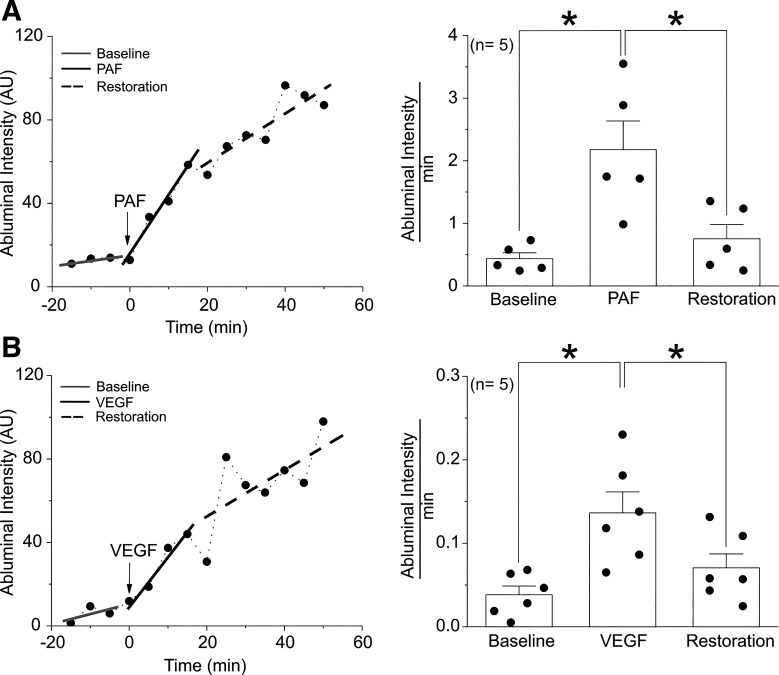
Endothelial barrier restoration is self-limiting. Human microvascular endothelial cells were grown to confluence and treated with platelet-activating factor (PAF) or vascular endothelial growth factor (VEGF) (*time point 0*). *A*, *left*: representative trace during a continuous 100 nmol/L PAF application initially increased the abluminal fluorescence intensity vs. time slope (an index of permeability), which subsequently declined, suggesting restoration toward control endothelial permeability. Representative data: baseline slope (−15 to −5 min) = 0.2897, PAF slope (0 to 15 min) = 2.888, and restoration slope (20 to 50 min) = 1.237. *A, right*: PAF slopes quantification *P < 0.05. *B*, *left*: representative trace during a continuous 1 nmol/L VEGF application initially increased the abluminal fluorescence intensity vs. time slope (an index of permeability), which subsequently declined, suggesting restoration toward control endothelial permeability. Representative data: baseline slope (−15 to −5 min) = 0.0466, VEGF slope (0 to 15 min) = 0.23, and restoration slope (20 to 50 min) = 0.1316. AU, arbitrary units. *B*, *right*: slopes quantification, *P < 0.05; *N* = 5.

To test the concept and to investigate the endothelial mechanisms of inactivation, we used two established in vitro systems to evaluate endothelial integrity and estimate the apparent solute permeability of the endothelial monolayer (2, 3, 14). Using the transwell permeability assay, we applied PAF (or VEGF) to HMVEC monolayers and evaluated the time course of the movement of FITC-dextran 70 kDa (FITC-Dx70) from the luminal to the abluminal compartment by measuring fluorescence intensity in arbitrary units (AU). Figure 1*A* demonstrates that 100 nmol/L PAF, applied throughout the entire experimental period, increases the transport of FITC-Dx70 across HMVEC monolayers, observed as an increase in abluminal fluorescence intensity. Notably, the abluminal fluorescence intensity versus time slope, an index of permeability, increases initially (AU = 2.888) and declines subsequently during PAF application (AU = 1.237), supporting the concept that endothelial barrier restoration is a self-limiting process. We detected similar patterns in HMVECs in response to 1 nmol/L VEGF stimulation (Fig. 1*B*).

The second method we used to evaluate endothelial permeability was the Electrical Cell-Substrate Impedance Sensing (ECIS) system, which is unique because it measures transendothelial electrical resistance (TER) of endothelial cell monolayers while providing a continuous time-course recording of changes in monolayer barrier properties under different treatment conditions. Supplemental Fig. S1 (all Supplemental Material is available at https://doi.org/10.6084/m9.figshare.21178975) shows changes in the TER of HUVECs during thrombin treatment. Note that in one case the administration of thrombin was maintained during the washout, whereas thrombin was removed during the washout in the other case. In both cases, TER initially decreases, indicating increased permeability, and subsequently returns toward baseline, indicating decreased permeability and an improvement in endothelial barrier function. The results seen with thrombin are consistent with the results seen with PAF and VEGF, two strong prohyperpermeability agonists (Fig. 1). These results provide strong evidence that the response that drives endothelial resistance/permeability back to baseline in the presence of the stimulus is independent of the applied agonist.

### Stimulation of Epac1 Inactivates Hyperpermeability In Vivo

Our previous results using endothelial cells indicate a physiological process associated with hyperpermeability inactivation (14). This observation suggests that the microcirculation possesses intrinsic mechanisms that trigger the return to the baseline to maintain microvascular and tissue homeostasis. Therefore, we evaluated the potential cellular mechanisms associated with hyperpermeability inactivation.

**Figure 2.**
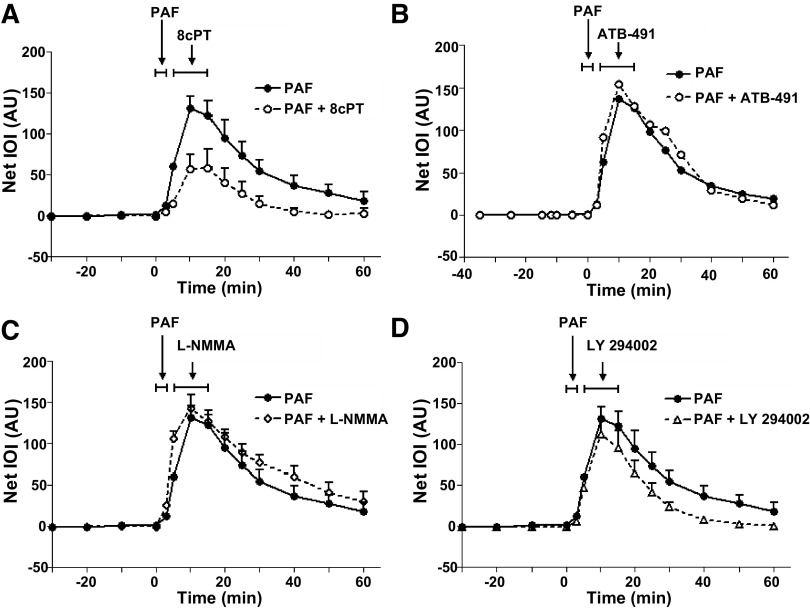
Stimulation of Epac-1 inactivates hyperpermeability in vivo. *A*: 100 nmol/L platelet-activating factor (PAF) induced a significant increase in permeability in mouse cremaster muscle, as indicated by an elevation in integrated optical intensity (IOI). Administration of 10 µmol/L 8cPT-cAMP (8cPT) after PAF challenge significantly reduced the peak IOI and accelerated the return of baseline permeability: *N* (PAF) = 5; *N* (PAF + 8cPT) = 5; *P* < 0.05 PAF vs. PAF + 8cPT at 10 to 50 min. *B*: application of 1 nmol/L ABT-491 (PAF receptor antagonist) after PAF administration failed to inactivate PAF-induced hyperpermeability: *N* (PAF) = 5; *N* (PAF + ABT-491) = 5. *C*: blocking eNOS production with 10 µmol/L l-NMMA (global NOS inhibitor) after PAF challenge failed to decrease the peak IOI: *N* (PAF) = 7; *N* (PAF + l-NMMA) = 5. *D*: application of 10 nmol/L LY-294002 (inhibitor of PI3K) after PAF challenge did not inhibit PAF-induced hyperpermeability: *N* (PAF) = 7; *N* (PAF + LY-294002) = 4. Data are shown as means ± SE. AU, arbitrary units.

The integrity of the endothelial barrier is largely regulated via the cAMP-Epac1-Rap1 axis, which controls the assembly of interendothelial junctional proteins (14–29). Thus, we tested whether Epac1 activation influences the hyperpermeability response in an in vivo mouse microvascular preparation. To evaluate the hyperpermeability in vivo, we used an established method using FITC-Dx70 as a macromolecular tracer (19, 20). We stimulated the in vivo mouse cremaster microvasculature topically for 3 min with 100 nmol/L PAF and applied 10 µmol/L 8cPT-cAMP (an Epac1 selective agonist) for 10 min, starting its application 2 min after the end of PAF administration (Fig. 2*A*). The idea was to selectively activate Epac1 during the upslope of hyperpermeability, i.e., immediately after its onset. PAF significantly increased permeability as indicated by the elevation and peak in integrated optical intensity (Fig. 2*A*). Application of 8cPT-cAMP after PAF challenge to the mouse cremaster significantly reduced the peak IOI and accelerated the return to baseline permeability (Fig. 2*A*). Using the hamster cheek pouch as another in vivo hyperpermeability microvascular model, we detected that stimulation of Epac1 accelerates the endogenous inactivation process as IOI returns promptly to baseline after Epac1 stimulation. Analysis of the peak IOI values demonstrates that 8cPT-cAMP applied 2 min after the end of 100 nmol/L PAF administration significantly inactivates hyperpermeability (Supplemental Fig. S2).

To assess whether or not inactivation of hyperpermeability depends on mechanisms related to triggering its onset, we tested first whether blocking PAF receptors shortly after the onset of hyperpermeability would inactivate the process. We applied 1 nmol/L ABT491, a PAF receptor antagonist, topically to the cremaster muscle 2 min after the end of the 3-min PAF administration. Blocking PAF receptors shortly after the onset of the rise in permeability did not inactivate PAF-induced hyperpermeability (Fig. 2*B*). Subsequently, we applied 1 nmol/L ABT-491 for 10 min before PAF application. Administration of ABT-491 before PAF blocked the onset of PAF-induced hyperpermeability completely (Supplemental Fig. S3). We hypothesized that administration of *N*^G^-monomethyl-l-arginine (l-NMMA) (global NOS inhibitor) could block cytosolic eNOS production and therefore terminate hyperpermeability. We tested the hypothesis by administering 100 µmol/L l-NMMA topically to the cremaster muscle 2 min after the end of the 3-min PAF administration. PAF as well as PAF followed by l-NMMA elevated IOI to a similar peak within ∼10 min and returned to baseline with similar time course (Fig. 2*C*). We also explored whether blockade of PI3K (with its inhibitor LY-294002) reduces the peak of IOI in response to PAF. Application of LY-294002 for 10 min (starting 2 min after PAF) changed neither the upslope nor the time course of the hyperpermeability response (Fig. 2*D*). We demonstrated previously that 10-min application of l-NMMA and of LY-294002 before the agonist (VEGF) was efficient to block hyperpermeability (25). These results highlight the importance of recognizing that PAF (and possibly other agonists) quickly triggers a rapid signaling cascade that determines the onset of hyperpermeability. Importantly, these results support the concepts that inactivation of hyperpermeability is a process separate from its activation, and that an agonist and/or the signaling cascade triggered by the onset of hyperpermeability may stimulate the delayed signaling by factors that cause the inactivation of agonist-induced hyperpermeability. In addition, these results suggest that activation and triggering of the inactivation cascade of hyperpermeability occur shortly after the arrival of the agonist to its receptor, inasmuch as blocking key elements after PAF application did not alter the time course of the response.

### Stimulation of Epac1 Inactivates Hyperpermeability in HMVEC Monolayer

**Figure 3.**
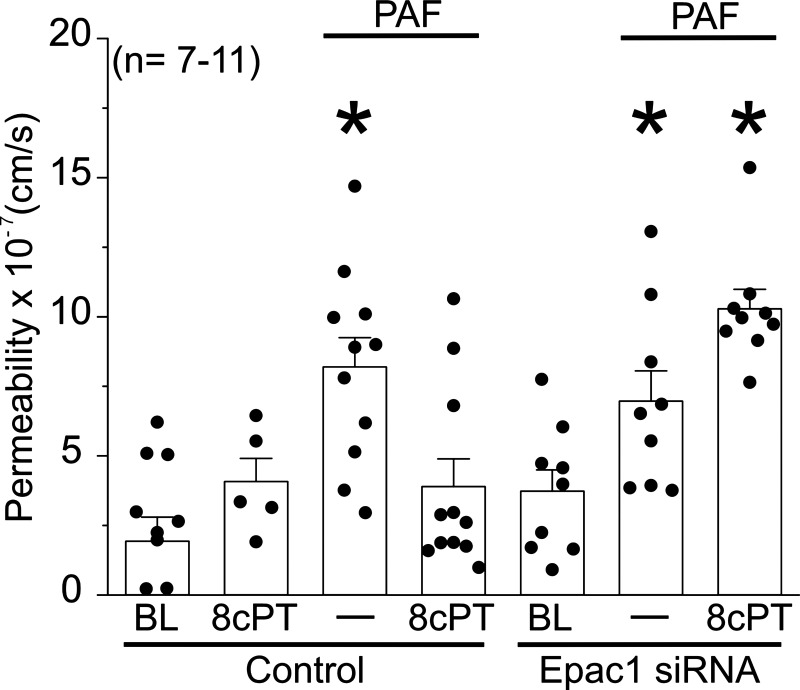
Stimulation of Epac1 inactivates agonist-induced hyperpermeability. Platelet-activating factor (PAF) treatment significantly increased human microvascular endothelial cell (HMVEC) monolayer permeability to FITC-Dx70 compared with baseline control (BL). Epac1 was stimulated with 8cPT-cAMP (8cPT). Application of 8cPT-cAMP alone did not cause significant changes in permeability. 8cPT- cAMP application after PAF strongly inactivated PAF-induced hyperpermeability. 8cPT-cAMP failed to block PAF-induced hyperpermeability in HMVECs depleted of Epac1 via siRNA. *P < 0.05 compared with BL, 8cPT, and PAF + 8cPT in control and compared with BL in Epac1 siRNA results. *N* = 9 in control and Epac1 siRNA.

To confirm our in vivo results described above, and to avoid any other possible secondary components playing a role in hyperpermeability inactivation such as nerves, adipocytes, muscle cells, etc., we used the transwell assay to test whether stimulation of Epac1 would trigger a reduction of agonist-induced hyperpermeability in vitro in HMVEC monolayers (Fig. 3*A*). We used 8cPT-cAMP as a selective agonist for Epac1. PAF significantly increased monolayer permeability to FITC-Dx70, whereas the application of 10 µmol/L 8cPT-cAMP alone did not cause significant changes in permeability, compared with baseline control. However, when 8cPT-cAMP was applied after PAF challenge, hyperpermeability was strongly inactivated. 8cPT-cAMP also inactivated the hyperpermeability caused by VEGF in HMVEC monolayers (Supplemental Fig. S4), showing that Epac1 stimulation inactivates hyperpermeability irrespective of the proinflammatory agonist.

We applied loss of function approach to confirm that Epac1 mediates the inactivation of PAF-induced hyperpermeability. We depleted HMVEC monolayers of Epac1 via small interfering (si)RNA (Epac1 siRNA 200 nM, 72 h transfection time, Supplemental Fig. S5). Cells transfected with Epac1-siRNA showed significant increase in permeability in response to PAF; however, they were insensitive to application of 8cPT-cAMP after PAF, and permeability remained elevated throughout the course of the experiment (Fig. 3). We corroborated our siRNA observations using pharmacological inhibition of Epac1 with ESI-09, a selective Epac inhibitor (30). ESI-09 inhibited Epac1 and prevented 8cPT-cAMP-stimulated inactivation, so that permeability remained elevated after PAF application (Supplemental Fig. S6). These data support the novel concept that inactivation of agonist-induced microvascular hyperpermeability is an active signaling process triggered by proinflammatory agents in a self-limiting manner, chiefly regulated through the endothelial cAMP-Epac1 pathway.

### Intracellular cAMP Regulates Endothelial Hyperpermeability Inactivation

**Figure 4.**
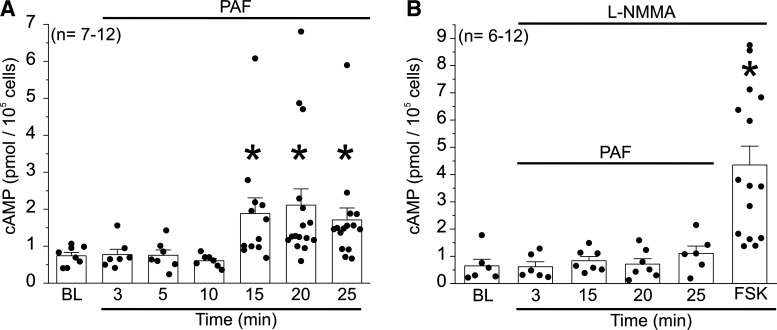
Platelet-activating factor (PAF) induces NO-dependent delayed increase in cAMP in human microvascular endothelial cells (HMVECs). *A*: treatment of HMVECs with 100 nmol/L PAF for 15, 20, and 25 min significantly increased cAMP concentration compared with baseline (BL). PAF administration for 3, 5, and 10 min did not change cAMP levels. *B*: pretreatment of HMVECs with *N*^G^-monomethyl-l-arginine (l-NMMA; global NOS inhibitor) completely blocked PAF-induced increase in cAMP. Forskolin (FSK) was used as positive control. *P < 0.05 compared with baseline; *N* = 7–12.

Epac1 activation is associated with an increase in intracellular endothelial cAMP concentration ([cAMP]) (16). Consequently, we evaluated the cAMP increase in our next experiments. Since our previous results support a role for endothelial cAMP in the inactivation of ischemia/reperfusion induced hyperpermeability in vivo and in vitro (14), we examined whether PAF elicits a [cAMP] response in HMVECs and measured its time course. Administration of 100 nmol/L PAF to HMVECs triggered a twofold significant increase in [cAMP] after 15, 20, and 25 min compared with baseline values. (Fig. 4*A*). Note that PAF administration for 3, 5, and 10 min did not change cAMP levels, suggesting that the timing of this cAMP response is consistent with the timing and concept of hyperpermeability inactivation (Figs. 1 and 2*A*) and that increased [cAMP] is a contributing factor in the self-limiting mechanism for agonist/cascade-induced endothelial hyperpermeability.

The onset of hyperpermeability requires production of eNOS-derived NO in the cytosol (3–5). It is plausible that the increase in cytosolic [NO] may also participate in triggering the delayed inactivation process. We tested this potential role of eNOS-derived NO by administering l-NMMA as a global NOS inhibitor. Inhibition of eNOS completely blocked PAF-induced increase in cAMP concentration (Fig. 4*B*). Under the same experimental conditions, 100 nmol/L forskolin (FSK), an activator of adenylyl cyclase, promotes an increase of cAMP (Fig. 4*B*). These results are strong evidence that the onset of hyperpermeability and its inactivation are linked by a novel pathway that connects NO production and cAMP synthesis.

### Epac1 Stimulation Induces Cytosol-to-Membrane eNOS Translocation

**Figure 5.**
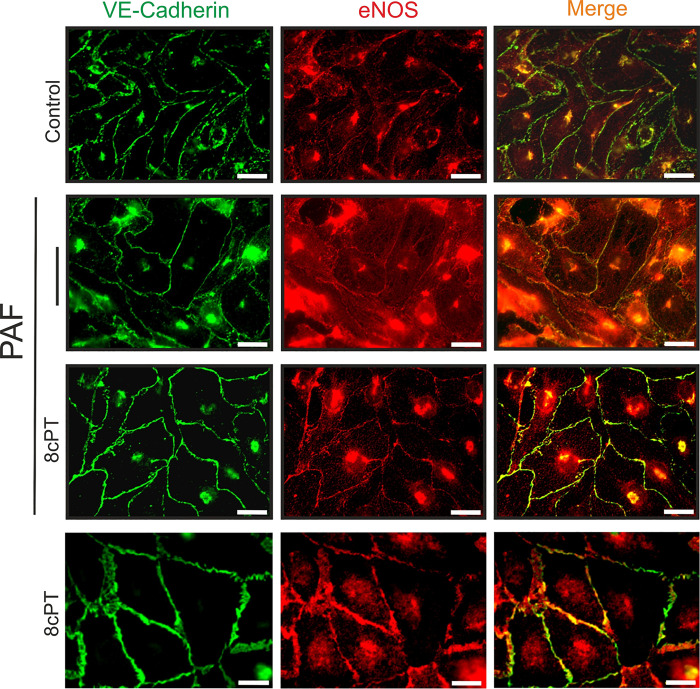
Epac-1 stimulation after platelet-activating factor (PAF) induces endothelial nitric oxide synthase (eNOS) movement from cytosol to plasma membrane. Representative immunofluorescence microscopy images of eNOS (red) and VE-cadherin (green) in confluent human microvascular endothelial cell (HMVEC) monolayers treated with PAF or PAF followed by 8cPT-cAMP (8cPT). In control cells, eNOS and VE-cadherin are present mainly in the cell membrane. PAF triggered the movement of eNOS from the plasma membrane to the cytosol, and loss of junctional integrity as indicated by “gaps” in VE-cadherin staining. Treatment with 8cPT-cAMP after PAF restored VE-cadherin integrity and translocated eNOS from the cytosol back to the cell membrane. 8cPT alone did not change eNOS distribution in endothelial cells. Scale bar = 20 µm.

In the absence of inflammatory stimuli, eNOS resides preferentially in cell membrane caveolae and at the Golgi complex. We demonstrated that the onset of agonist-induced hyperpermeability requires eNOS internalization to the cytosol (4, 5). We also showed in vivo that eNOS location at the Golgi is associated with the onset of vasodilation (31). Because subcellular eNOS location is intrinsically linked to its vascular function, we hypothesized that inactivation of hyperpermeability entails a mechanism that enables the return of eNOS from the cytosol to the cell plasma membrane. We tested this hypothesis by immunofluorescence microscopy using VE-cadherin as marker for cell membrane. Figure 5 shows indirect immunocytochemical staining to observe the location of eNOS and VE-cadherin. We challenged confluent HMVECs with PAF, or with PAF for 3 min followed by 8cPT-cAMP for 10 min (without removing PAF). PAF triggered the mobilization of eNOS from the plasma membrane to the cytosol, concomitant with loss of junctional integrity as indicated by VE-cadherin staining. Application of 8cPT-cAMP after a 3-min PAF challenge led to the visualization of eNOS at the cell plasma membrane. 8cPT-cAMP alone (applied for 10 min) did not alter eNOS subcellular location (Fig. 5). This result suggests that the cAMP-Epac1 axis mediates the inactivation of endothelial hyperpermeability by promoting the translocation of eNOS from the cytosol back to the cell membrane. To our knowledge, this is the first demonstration of eNOS retro-translocation to the plasma membrane is linked to vascular function. Thus, our data further confirm the concept that eNOS translocation is a central signaling module in the control of microvascular barrier function during acute inflammation.

### Proinflammatory Agonists Stimulate Close Localization of Epac1 and eNOS in HMVECs

**Figure 6.**
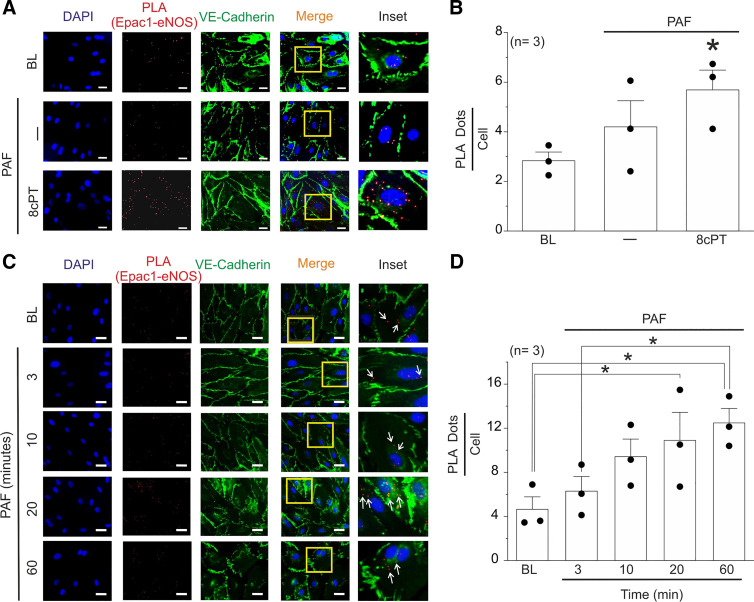
Epac1 and endothelial nitric oxide synthase (eNOS) are associated in endothelial cells. *A*: representative images obtained by proximity ligation assay (PLA) of the spatial association between Epac1 and eNOS (red dots) in human microvascular endothelial cells (HMVECs) at baseline, after 100 nmol/L platelet-activating factor (PAF) for 3 min, and pretreated with 8 cPT-cAMP (8cPT) for 10 min. Green fluorescent signal corresponds to VE-cadherin (membrane) and the blue signal to DAPI (nuclei). *B*: quantification of the PLA-detected association between Epac1-eNOS observed in *A*. Note that the association between these two proteins is significantly higher after administration of 8cPT-cAMP. We used three independent endothelial cultures and analyzed 3/4 pictures per treatment. Values are means ± SE; *N* = 3. *P < 0.05 vs. baseline (BL). *C*: representative figures obtained by PLA of the spatial association between Epac1 and eNOS (red dots) in HMVECs at baseline and with 100 nmol/L PAF for 3, 10, 20, and 60 min. Green fluorescent signal corresponds to VE-cadherin (membrane) and the blue signal to DAPI (Nuclei). Arrows indicate Epac1-eNOS proximity. *D*: proximity analysis of the PLA-detected association between Epac1-eNOS observed in *C*. Note that the association between these two proteins is significantly higher after 20 min of PAF stimulation. We used three independent endothelial cultures and analyzed 3/4 pictures per condition. Values are means ± SE. ∗*P* < 0.05 vs. BL. Scale bar = 20 μm.

Our results indicate that Epac1 activation restores eNOS localization to the cell plasma membrane inactivating the hyperpermeability process. We tested the localization of eNOS and Epac1 through proximity ligation assay (PLA) (32). We used VE-cadherin and DAPI as cell membrane and nucleus markers, respectively. Epac1-eNOS interaction is visualized as red dot stains (Fig. 6, *A* and *B*). In baseline and after 3 min of 100 nmol/L PAF stimulation, Epac1/eNOS interaction revealed similar red dots/cell values. Strikingly, cells pretreated with 8cPT-cAMP for 10 min show higher protein interactions between Epac1/eNOS than baseline, indicating that PKA activation promotes proximity between these proteins. Furthermore, after 20 min of PAF application, we detected a twofold signal increase in protein interactions relative to baseline. A similar pattern was detected following 60 min of PAF administration, supporting the concept that Epac1 interacts with eNOS throughout the course of inactivation of agonist-induced hyperpermeability (Fig. 6, *C* and *D*).

### PAF-Induced Retro-Translocation of eNOS from Cytosol to Plasma Membrane Requires eNOS-Derived NO and VASP Phosphorylation

Based on established intracellular trafficking mechanisms, we postulated that eNOS translocation to the plasma membrane is aided by an actin-regulating protein. Among the numerous proteins that interact with and regulate actin, vasodilator-stimulated phosphoprotein (VASP) is a strong candidate for this role as it regulates actin polymerization and contributes to cell adhesion and barrier integrity (6, 33). Indeed, deletion of the genes encoding for the Ena (Mena)-VASP protein family leads to embryonic death largely because of edema (33). VASP is involved in the control of endothelial barrier integrity through its role in the assembly of focal adhesions (27, 33). Because of its important role in regulating barrier integrity, we hypothesized that VASP may be a significant mediator of the agonist-initiated inactivation of hyperpermeability.

**Figure 7.**
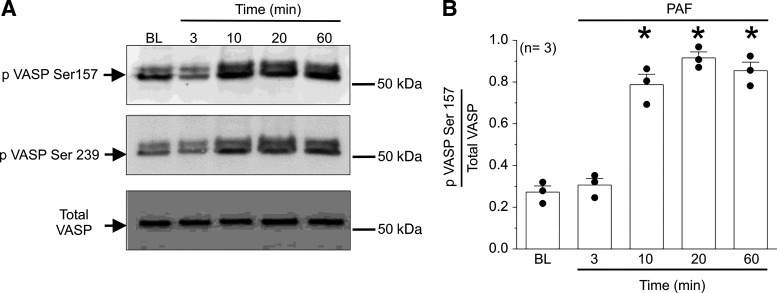
Platelet-activating factor (PAF) elicits vasodilator-stimulated phosphoprotein (VASP) phosphorylation at Ser157 and Ser239. MyEnd cells monolayers were treated with PAF for 3, 10, 20, and 60 min. *A*: VASP phosphorylation at Ser157 and Ser239 was assessed using Western Blot analysis. *B*: quantification of the phosphorylation of Ser157 from multiple Western blots. Phosphorylation of Ser157 was significantly higher than baseline (BL) for 10, 20, and 60 min after PAF administration. *P < 0.05 vs. BL control; *N* = 3.

VASP function and activity is critically regulated by phosphorylation of two serine (Ser) residues that are highly conserved sites, Ser157 and Ser239 (27). We tested the ability of PAF to elicit VASP phosphorylation at Ser157 and Ser239. We used MyEnd cells (wild type, WT), which are immortalized microvascular EC isolated from the mouse myocardium. In MyEnd WT monolayers, PAF stimulated VASP phosphorylation at both sites. Phosphorylation at Ser157, which is mainly caused by PKA, was significantly different from control at 10, 20, and 60 min after PAF application (Fig. 7). In addition, PAF triggered similar results at Ser239, usually caused by PKG, at the same time points described for Ser157. VASP phosphorylation at Ser157 and Ser239 is consistent with the termination of hyperpermeability. Moreover, this phenomenon is independent of the proinflammatory agonist used, as 1 nmol/L VEGF displayed similar results on VASP phosphorylation (Supplemental Fig. S7, *A* and *B*). To support the results seen in MyEnd cells, we tested VASP phosphorylation at Ser157 and Ser239 in HMVECs treated with 100 nmol/L PAF. We detected increased phosphorylation at both Ser157 and Ser239 on treatment with PAF (Supplemental Fig. S7*C*). Overall, our results suggest that VASP phosphorylation promoted by proinflammatory agonists in microvascular endothelial cells may be a step in the signaling pathway leading to the timed inactivation of hyperpermeability.

Because the onset of agonist-induced hyperpermeability requires eNOS-derived NO, and our hypothesis requires an interaction between NO and inactivation, we tested whether PAF triggers VASP phosphorylation in the absence of eNOS-derived NO. We applied 10 µmol/L l-NMMA to inhibit eNOS activity. Inhibition of eNOS-derived NO synthesis prevented PAF-induced VASP phosphorylation at Ser157 and Ser239 indicating that PAF-induced NO production precedes PAF-induced VASP phosphorylation (Supplemental Fig. S8). Together, our data support the novel concept that VASP phosphorylation in EC depends on proinflammatory agonist challenge and NO production.

### Epac1-Mediated Inactivation of Hyperpermeability Requires VASP

**Figure 8.**
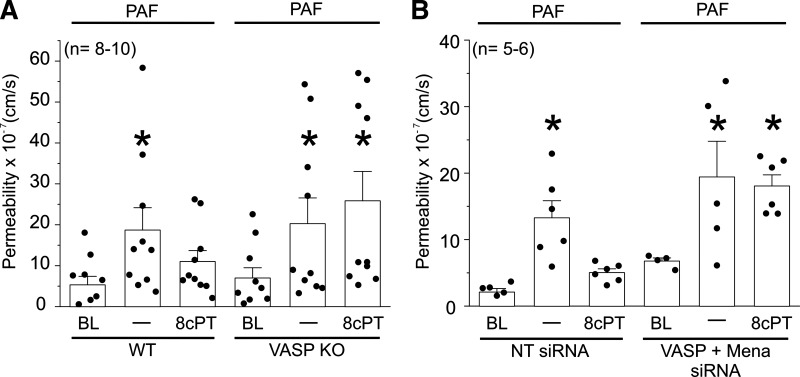
Epac1-mediated inactivation of hyperpermeability requires vasodilator-stimulated phosphoprotein (VASP). *A*: stimulation of Epac1 in VASP-knockout (KO) mouse myocardial endothelial (MyEnd) cells fails to inactivate platelet-activating factor (PAF)-induced hyperpermeability. MyEnd wild-type (WT) and VASP-KO cells monolayers were treated with PAF or PAF followed by 8cPT-cAMP (PAF + 8cPT). PAF significantly increased the permeability of MyEnd WT cells, which was readily inactivated by Epac1 stimulation with 8cPT. MyEnd VASP-KO cells showed hyperpermeability to PAF but failed to show inactivation of hyperpermeability on treatment with 8cPT. *P < 0.05 vs. baseline (BL, WT, and VASP-KO) and PAF + 8cPT (WT); *N* = 6. *B*: VASP depletion inhibits Epac1-induced inactivation of hyperpermeability in human endothelial cells. HMVECs transfected with nontargeting (NT) siRNA or VASP/Mena siRNA were treated with PAF or PAF followed by 8cPT (PAF + 8cPT). PAF caused significant hyperpermeability in both NT and VASP/Mena siRNA cells. However, stimulation of Epac1 with 8cPT after PAF failed to inactivate hyperpermeability in VASP/Mena-depleted cells. *P < 0.05 vs. baseline (BL in NT siRNA and VASP + Mena siRNA) and PAF + 8cPT in NT siRNA; *N* = 10.

Based on our observations that Epac1 mediates the inactivation of hyperpermeability and that VASP is phosphorylated by PAF/VEGF in a NO-dependent manner, we investigated whether VASP is required for Epac1-mediated hyperpermeability inactivation. For these studies, we used MyEnd WT cells, and MyEnd VASP-KO cells derived from VASP-KO mice (15, 16, 34). MyEnd WT cells showed a robust response to PAF, which was readily inactivated by stimulation of Epac1 with 8cPT-cAMP (Fig. 8*A*). In contrast, MyEnd VASP-KO cells showed augmented hyperpermeability to PAF, but failed to show inactivation of hyperpermeability on stimulation of Epac1 with 8cPT-cAMP. Note that the expression levels of other endothelial proteins involved in the hyperpermeability such as eNOS, VE-cadherin, and p120-catenin were not affected in MyEnd VASP-KO cells (Supplemental Fig. S7*D*). Therefore, these results display the key role of VASP in hyperpermeability inactivation. Overall, our data indicate that VASP is a necessary assisting element in the cAMP-Epac1-mediated pathway that leads to hyperpermeability inactivation.

To confirm the results obtained with MyEnd VASP-KO cells in primary EC, we tested whether human EC depleted of VASP via siRNA were able to efficaciously respond to pharmacological Epac1 stimulation. We transfected HMVECs with nontargeting siRNA duplexes (control), or with siRNA targeting VASP. To minimize possible compensatory adaptation by members of the Ena/VASP (Mena in mammals) family of actin regulators, we performed a VASP/Mena double depletion in HMVECs and subsequently applied PAF or PAF followed by 8cPT-cAMP (Fig. 8*B*). HMVEC monolayers depleted of VASP/Mena did not show significant changes in baseline permeability compared with nondepleted control cells and showed a robust response to PAF. However, 8cPT-cAMP failed to inactivate hyperpermeability after PAF in VASP/Mena-depleted cells, recapitulating our observations in MyEnd VASP-KO cells. In summary, these data demonstrate that Epac1-mediated inactivation of hyperpermeability requires the expression and activity of VASP/Mena proteins in human and mouse microvascular EC.

### VASP Is Necessary for the Translocation of eNOS Back to the Endothelial Cell Membrane

**Figure 9.**
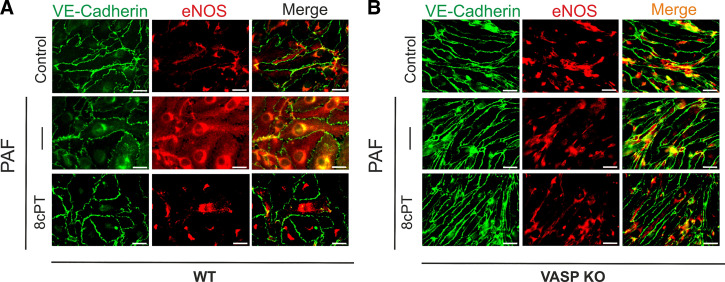
Vasodilator-stimulated phosphoprotein (VASP) is necessary for endothelial nitric oxide synthase (eNOS) translocation from cytosol to the cell membrane. Representative images of confluent wild-type (WT) or VASP-knockout (KO) myocardial endothelial (MyEnd) cells. *A*: eNOS (red) is localized at the cell membrane (identified by VE-cadherin, green) in control WT cells. Platelet-activating factor (PAF)-treated cells show less eNOS at the cell membrane and increased eNOS in the cytosol. Treatment with 8cPT-cAMP after PAF induced the reappearance of eNOS at the cell membrane. *B*: eNOS localizes mainly to the cytosol in control MyEnd VASP-KO cells. PAF did not alter eNOS distribution. eNOS remained in the cytosol following application of 8cPT-cAMP after PAF. We used three independent endothelial cultures and analyzed 3/4 pictures per treatment. Scale bar = 20 µm.

Since VASP controls actin dynamics and is involved in the assembly of junctional complexes in EC, we investigated whether VASP is involved in the Epac1-associated translocation of eNOS from the cytosol back to the cell membrane. We exposed MyEnd WT and MyEnd VASP-KO monolayers to PAF, 8cPT-cAMP, or PAF followed by 8cPT-cAMP and assessed the cellular distribution of eNOS and VE-cadherin. PAF triggered eNOS internalization in WT cells (Fig. 9*A*). Application of PAF followed by 8cPT-cAMP led to the translocation of eNOS back to the plasma membrane of WT cells (Fig. 5). In contrast, eNOS distribution in MyEnd VASP-KO cells was largely cytosolic. This distribution was not altered by application of PAF. eNOS remained in the cytosol even after application of 8cPT-cAMP in VASP-KO cells (Fig. 9*B*). These results confirm that VASP is a crucial protein in the regulation of eNOS translocation, which plays a vital role in the hyperpermeability inactivation processes.

## DISCUSSION

We provide evidence in support of the novel hypothesis that inactivation of microvascular hyperpermeability is an active signaling process intrinsic to endothelial cells and is triggered by the agonist(s) causing hyperpermeability. We demonstrate that *1*) PAF and VEGF, in addition to causing hyperpermeability, stimulate the cAMP/Epac1 pathway with a time-delay consistent with agonist-induced inactivation of hyperpermeability; *2*) inactivation of hyperpermeability is associated with eNOS movement from the cytosol to the EC membrane; *3*) eNOS movement from cytosol to cell membrane is associated with cAMP activation of Epac1; and *4*) VASP, an actin-regulating protein, is required for the translocation of eNOS to the cell membrane, and is necessary for the inactivation of hyperpermeability.

### Inactivation of Hyperpermeability Is Triggered by an Endothelial Negative Feedback Mechanism

We propose that the timed inactivation of hyperpermeability results from a series of biochemical events triggered by the agonist that elicits the onset of hyperpermeability. This delayed inactivation response constitutes intrinsic negative feedback that operates in ECs to maintain vascular and tissue homeostasis while sustaining the hyperpermeability state required for the repair of injured tissue. Our in vitro and in vivo data indicate that a 3-min PAF pulse suffices to mount a robust hyperpermeability response, whereas the start of the downslope associated with inactivation is observed at ∼10–15 min after PAF challenge (14) (Fig. 2). Importantly, hyperpermeability returns to baseline whereas the agonist is present in vivo and in vitro (1, 2). Such results are supported by our data demonstrating that the return to baseline is independent of the presence of the agonist. Consequently, it seems unlikely that inactivation is triggered simply by the removal of the agonist, supporting the concept that hyperpermeability and endothelial barrier restoration is a self-limiting process.

PAF-induced hyperpermeability operates by activating a signaling cascade that causes eNOS translocation to the cytosol as well as eNOS phosphorylation and NO production and leads to *S*-nitrosylation of junctional proteins (2–7). We postulate that proinflammatory agonist (i.e., PAF or VEGF), through its stimulated production of eNOS-derived NO, causes a delayed second cascade that initiates and accomplishes inactivation of hyperpermeability and restores endothelial barrier properties. Our hypothesis implies that elevated cytosolic NO concentration causes a change in the factors associated with maintenance of endothelial barrier properties. In support of this requirement, we demonstrated that PAF elicits a delayed increase in [cAMP], which is mediated by NO, inasmuch as blockade of eNOS completely inhibits the PAF-stimulated increase in [cAMP] (Fig. 4). The molecular events that link NO production and the delayed cAMP synthesis response are beyond the scope of our study and remain to be elucidated.

Our previous studies on endothelial/hyperpermeability homeostasis focused on the regulation of proteins/signaling pathways affecting barrier function through the paracellular pathway. In this regard, the current work advances our studies by evaluating mechanisms associated with hyperpermeability inactivation. We did not perform experiments to test possible contributions via transcellular pathways.

### cAMP-Epac1 Axis Regulates Inactivation of Hyperpermeability

Maintenance of endothelial barrier properties by cAMP operates via Epac1 and activates Rap1 (13, 14, 16, 22, 29, 35). Our hypothesis includes triggering of this signaling pathway to promote inactivation of hyperpermeability. Before the development of our hypothesis, most studies sought to prevent hyperpermeability by applying inhibitors or agents capable of enhancing barrier function before the administration of proinflammatory factors. To better mimic clinical situations and to investigate the role of this signaling pathway in the inactivation of hyperpermeability, we targeted the cAMP-Epac1 axis after the onset of hyperpermeability. We applied both gain and loss of function approaches to test our hypothesis. As gain of function, we stimulated Epac1 using 8cPT-cAMP—a selective agonist for Epac1 (29). Stimulation of Epac1 with its selective agonist 8cPT-cAMP effectively inactivated hyperpermeability induced by PAF both in vivo and in vitro (Figs. 2 and 3). Such results were replicated when hyperpermeability was induced with VEGF, showing that Epac1 is involved in the restoration of barrier function independently of the inflammatory challenge (Supplemental Fig. S4). We did not test for Rap1 activation as we and others have previously shown that Epac1 interacts with Rap1 leading to restoration of barrier properties (13, 14).

Because it is plausible that removal of the agonist may passively cause the return of permeability to baseline, we tested in vivo whether inhibiting key steps associated with onset of hyperpermeability would alter the time course of the response to PAF. Our first test consisted of blocking PAF receptors with ABT-491. In addition, we blocked eNOS after PAF application with l-NMMA, and we applied LY-294002 to block PI3K and Akt signals upstream of eNOS phosphorylation and NO production. In all cases, we applied the inhibitors for 10 min, following 3 min of PAF application, as in the experiments with 8cPT-cAMP. This is the approximate time between the start of the rise in permeability and its peak in the cremaster microvasculature. Our results demonstrate that the inhibitors did not alter the time course of the hyperpermeability response to PAF (Fig. 2, *B**–D*). However, the 10-min period of application is sufficient to achieve blockade of the hyperpermeability response when the inhibitor is applied before the permeability enhancing agonist (14) (Supplemental Fig. S3). Our results demonstrate that PAF triggers the onset of hyperpermeability in the initial 3 min of its in vivo administration and that immediately subsequent inhibition of its receptors or its stimulated NO production does not alter the time course of the hyperpermeability response and its inactivation. These results support our hypothesis that inactivation of microvascular hyperpermeability is a self-limiting process that is triggered by the agonist. The timing of PAF-initiated in vivo hyperpermeability matches measurements of eNOS phosphorylation and NO production in endothelial cells (3, 4, 36), the *S*-nitrosylation of junctional proteins (2) and measurements of hyperpermeability in isolated venules (37).

To examine the role of Epac1 signaling in the inactivation of hyperpermeability via loss of function, we depleted Epac1 via siRNA and inhibited it with ESI-09, a global Epac inhibitor. Both approaches blocked the inactivation of PAF-induced hyperpermeability (Fig. 3; Supplemental Fig. S6), complementing and advancing other reports showing that Epac1 participates in the restoration of the endothelial barrier (16, 22, 29). Importantly, our results indicate that inactivation of hyperpermeability, while linked to the original stimulus, proceeds by triggering a separate signaling cascade. This conclusion is strongly supported by the failure to alter the time course of PAF-induced hyperpermeability through blocking PAF receptors and key elements of eNOS-derived NO production during the onset of hyperpermeability. Our data also support the efficacy and importance of the cAMP-Epac1 axis in inactivation of hyperpermeability.

We also investigated the signaling cascade involved in the inactivation process and its relationship to the cAMP-Epac1 axis. Previous studies had shown that cAMP stimulates Epac1 activation in endothelial cells (38). Given the role of the cAMP-Epac1 axis in the inactivation of hyperpermeability, we sought to investigate whether stimulation with PAF would influence intracellular cAMP levels. Our in vitro data show that there is a delayed increase in [cAMP] following PAF treatment, which starts around 15 min after PAF application (Fig. 4*A*). This increase in [cAMP] coincides with the downslope of hyperpermeability shown in Fig. 2, supporting the involvement of this signaling pathway in the intrinsic ability of the endothelium to terminate agonist-induced hyperpermeability. Importantly, we elucidated that NO production during the onset of agonist-induced hyperpermeability is linked to the inactivation of hyperpermeability via the cAMP-Epac1 axis, as blockage of NOS inhibited the delayed increase in [cAMP] on treatment with PAF (Fig. 4*B*).

### Inactivation of Hyperpermeability Involves VASP-Mediated Retro-Translocation of eNOS from Cytosol to Cell Membrane

We and others have established the fundamental role played by eNOS movement and localized NO delivery to subcellular targets; in particular, the movement from cell membrane to cytosol (3, 8, 36). In this work, we demonstrate that stimulation of the cAMP/Epac1 pathway with 8cPT-cAMP at times consistent with the onset of inactivation leads to the return of eNOS from the cytosol back to the EC cell membrane (Fig. 5). Therefore, we postulated that the cAMP/Epac1 pathway must trigger a trafficking event that leads to the return of eNOS to its cell membrane location. In support of this concept, we show that Epac1 stimulation following PAF treatment leads to an increase in the cytosolic proximity between Epac1 and eNOS (Fig. 6, *A* and *B*), which is consistent with the timing of inactivation of hyperpermeability (Fig. 6, *C* and *D*). Because of evidence that actin contributes to protein trafficking in endothelial cells (39, 40), we tested the involvement of VASP in the inactivation of hyperpermeability and in the Epac1-induced retro-translocation of eNOS to the cell membrane. VASP and its family members Mena and Ena/VASP like (EVL) are well-known regulators of the actin cytoskeleton. We provide evidence here that VASP is activated by PAF in a NO-dependent manner and that Epac1 stimulation requires VASP proteins to inactivate hyperpermeability and to return eNOS back to the cell membrane (Fig. 7; Supplemental Figs. S7 and S8). Interestingly, caveolin-1 (cav1) is a protein that associates with and inactivates eNOS (41, 42). Our data indicate that VASP phosphorylation, followed by its association with eNOS retro-translocation of eNOS back to the plasma membrane, precedes the inactivation of eNOS by Cav1.

Consistent with a delayed onset of inactivation of hyperpermeability, PAF induced significant phosphorylation of VASP 10 min after its application on Ser157 (Fig. 7 and Supplemental Fig. S7). Phosphorylation of VASP on Ser157 is usually stimulated via PKA, which is activated by cAMP (34). Interestingly, VASP also contributes to the onset of hyperpermeability (6). It is plausible that VASP’s contribution to the onset of hyperpermeability operates via PAF stimulation of VASP phosphorylation on Ser239, which was significant at all the times measured in this study. It is important to note that phosphorylation of VASP on Ser239 is usually caused by PKG, and that PKG activity is associated with cGMP. In addition, PAF-induced *S*-nitrosylation of VASP—specifically on Cys64—leads to hyperpermeability (6). We interpret our findings regarding the dual role of VASP as evidence of a retrieval mechanism intrinsic to the negative feedback that enables hyperpermeability inactivation. In the absence of inflammatory stimulation, VASP maintains endothelial barrier integrity (15). PAF stimulated synthesis of cytosolic eNOS-derived NO causes VASP *S*-nitrosylation and leads to barrier disruption. In a delayed manner, PAF and VEGF promote NO-dependent increase in cytosolic cAMP and VASP phosphorylation on Ser157 (Figs. 4 and 7, Supplemental Fig. S7, *A–C*), which contributes to eNOS membrane localization. In support of our interpretation, VASP membrane localization is largely accepted as being regulated by serine 157 phosphorylation (27).

The mechanisms that link Epac1 activation to VASP phosphorylation and translocation in endothelial cells are poorly understood. In hepatocytes undergoing bile acid-stimulated apicobasal polarization, cAMP/Epac1 leads to the membrane insertion of apical proteins via a complex signaling network that involves several kinases, such as MEK, LKB1, and AMPK (43). It is unclear whether these pathways operate in the vascular endothelium. However, as a highly polarized phenotype is crucial for the integrity of the endothelial barrier, it is possible that similar mechanisms act to sustain barrier function, and to restore it after inflammatory challenge.

**Figure 10.**
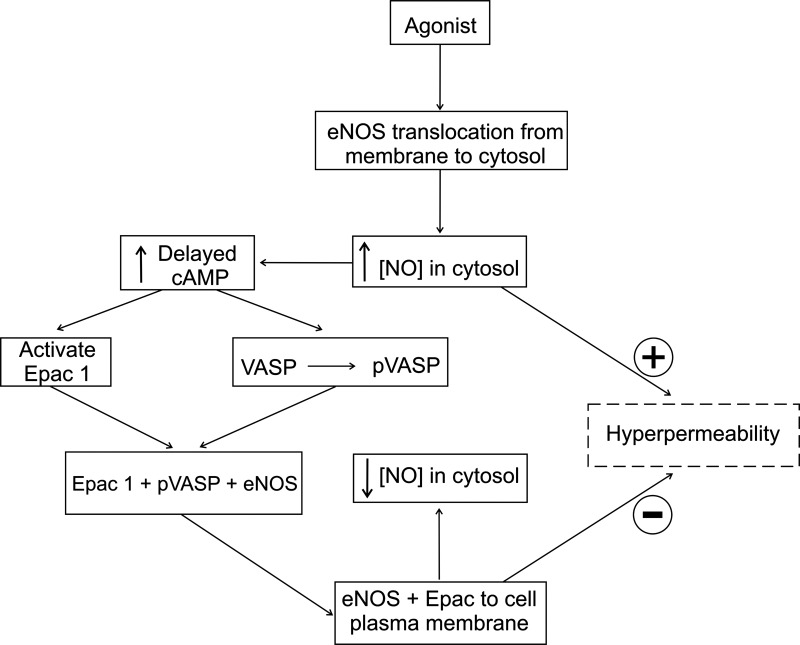
Proposed hypothesis: inactivation of hyperpermeability is an intrinsic self-limiting property of the microvascular endothelium that maintains vascular homeostasis during inflammatory conditions. Proinflammatory agonists mediate the translocation of endothelial nitric oxide (NO) synthase (eNOS) from the plasma membrane to the cytosol (box with dashed lines). The eNOS-derived NO increase in cytosol [NO] leads to hyperpermeability and delayed increase in cAMP concentration ([cAMP]). The increase in [cAMP] causes the phosphorylation of vasodilator-stimulated phosphoprotein (VASP) on Ser157 and Ser239, which assists in the retro-translocation of eNOS back to the cell membrane (boxes with solid lines). eNOS retro-translocation leads to inactivation of hyperpermeability.

In summary, our data indicate the existence of an endogenous, active pathway of inactivation of hyperpermeability, in which inflammatory agonists trigger a time-delayed increase in intracellular cAMP, leading to Epac1 activation and restoration of baseline permeability. Figure 10 illustrates our proposed hypothesis. Proinflammatory agonists (PAF, VEGF, etc.) initiate hyperpermeability to macromolecules through receptor-mediated mechanisms that translocate eNOS to the cytosol via caveolar internalization (2–4, 36). The elevated cytosolic concentration of eNOS-derived NO leads to hyperpermeability and to delayed increased [cAMP] (Fig. 4). In turn, delayed increase in [cAMP] causes phosphorylation of VASP (Fig. 7). We postulate that phosphorylation of VASP in ser157 assists in translocation of eNOS to the cell membrane (Fig. 9), contributing to inactivation of hyperpermeability.

To our knowledge, this is the first report of an endothelial self-limiting mechanism, which supports the novel hypothesis that proinflammatory agonists cause the onset of hyperpermeability as well as the onset of its inactivation. Our hypothesis is innovative because it frames the termination of vascular leakage as an intrinsic endothelial signaling pathway. Our findings also elucidate new pathways that may be potential pharmacological targets in the development of therapeutic interventions to terminate microvascular hyperpermeability. We anticipate that this study will produce additional advances leading to improved therapeutics that may influence the way in which physicians and vascular surgeons approach prevention and termination of edema and compartment syndrome resulting from prolonged hyperpermeability in inflammation, ischemia-reperfusion injury, and vascular disease.

## DATA AVAILABILITY

Data will be made available upon reasonable request.

## SUPPLEMENTAL DATA

10.6084/m9.figshare.21178975 Supplemental Figs. S1–S8: https://doi.org/10.6084/m9.figshare.21178975.

## GRANTS

This work was supported by National Heart, Lung, and Blood Institute Grant HL146539 (to W.N.D.); American Heart Association Career Development Award 932684 (to M.A.L.); and funds from Rutgers School of Graduate Studies (to A.P.T.).

## DISCLOSURES

No conflicts of interest, financial or otherwise, are declared by the authors.

## AUTHOR CONTRIBUTIONS

W.N.D. conceived and designed research; P.R.N., P.C.B., M.A.L., P.E.M., T.I., J.Z., R.G.D., M.B., N.G., D.D.K., N.G.A., J.W.B., and F.A.S. performed experiments; P.R.N., P.C.B., M.A.L., P.E.M., T.I., J.Z., R.G.D., N.G., N.G.A., J.W.B., F.A.S., and W.N.D. analyzed data; P.R.N., P.C.B., M.A.L., A.P.T., and W.N.D. interpreted results of experiments; P.C.B., M.A.L., and R.G.D. prepared figures; W.N.D. drafted manuscript; P.R.N., P.C.B., M.A.L., M.B., and W.N.D. edited and revised manuscript; P.R.N., P.C.B., M.A.L., P.E.M., T.I., J.Z., R.G.D., M.B., N.G., D.D.K., N.G.A., A.P.T., J.W.B., F.A.S., and W.N.D. approved final version of manuscript.
